# Supplementary material for: The genome sequence of Bird’s-foot, Ornithopus perpusillus L. (Fabales: Fabaceae)
Source: Wellcome Open Res. 2026 Sep 17;11:691. [Version 1] doi: 10.12688/wellcomeopenres.27834.1 (PMC13620360; doi:10.12688/wellcomeopenres.27834.1)
Supplement: Peer review history

# Reviewer report: Reviewer response for version 1

**DOI:** 10.21956/wellcomeopenres.30629.r170395

**Published:** 2026-09-28

**Authors:** Ruslan Kalendar

**Affiliations:** 1 University of Helsinki, Helsinki, Finland

This Genome Note reports a haplotype-resolved, chromosome-level assembly of *Ornithopus perpusillus*, produced by the Darwin Tree of Life pipeline from PacBio HiFi and Hi-C data. Haplotype 1 is 528.92 Mb, with 99.73% of it in seven chromosomal pseudomolecules, consistent with n = 7. It has a contig N50 of 3.66 Mb, QV 67.0, and BUSCO completeness of 97.4%. The note also reports mitochondrial and plastid genomes.

 Haplotype 1 is a good resource and clearly meets the EBP 6.C.Q40 standard. Most of my concerns are about internal consistency and how haplotype 2 is presented. I checked the core numbers, and they hold up.

 The total length of the chromosomes was 527.52 Mb, which is 99.73% of the assembly.

 As reported, N50 is chromosome 3, and N90 is chromosome 7.

 42 scaffolds plus 223 gaps gave the reported 265 contigs.

 The Assembly statistics section says haplotype 1 "was curated to chromosome level" while haplotype 2 "was also scaffolded." The Assembly curation section says the two haplotypes "were combined for curation." Please clarify whether haplotype 2 was manually curated or not. Separately, a 7.3% increase in the total assembly length from curation needs to be explained. The 61 breaks and 116 joins would add only tens of kb of gap sequence, not approximately 36 Mb. Was the sequence moved between haplotypes, or were the haplotypes reassigned?

 The flow cytometry estimate is 670 Mb and the GenomeScope estimate is 572 Mb. The assembly is 529 Mb, so approximately 21% of the cytometric genome size is missing. This is common and is most likely a collapsed satellite, centromeric, and rDNA repeat. A sentence addressing this would help, along with telomere and centromere status for each chromosome, which is not reported. The Hi-C map suggests good chromosome-scale structure, but readers can not tell whether any chromosomes are telomere-to-telomere.

 0.69 pg × 978 Mb/pg gives approximately 675 Mb, not 670.00 Mb. The two decimal places also imply precision, as a two-significant-figure pg value can not support.

 18.39 Gb of HiFi over 528.92 Mb is about 35×, but the text says about 30×. The blob plot places the main scaffolds near 20×.

****

Are the datasets clearly presented in a usable and accessible format, and the assembly and annotation available in an appropriate subject-specific repository?

Yes

Are sufficient details of the sequencing and extraction, software used, and materials provided to allow replication by others?

Yes

Are the rationale for sequencing the genome and the species significance clearly described?

Yes

Are the protocols appropriate and is the work technically sound?

Yes

Reviewer Expertise:

plant bioinformatics and genomics

I confirm that I have read this submission and believe that I have an appropriate level of expertise to confirm that it is of an acceptable scientific standard.

# Reviewer report: Reviewer response for version 1

**Published:** 2026-09-17

**Authors:** Automated Benchmarking

**Affiliations:** 1 Wellcome Sanger Institute, Hinxton, UK

As this article is a Genome Note, Automated Benchmarking has been used to determine whether the reported assembly passes four defined checks established by the Earth BioGenome Project: the raw and assembled data are publicly available, the Earth BioGenome Project three-part metric is met, the k-mer completeness exceeds 90%, and the BUSCO score passes the stated threshold. For Ornithopus perpusillus (drOrnPerp1), all four checks passed. Data available: pass. ENA study PRJEB80118, raw-read accessions ERR13698302, ERR13698301, and ERR13670062, and assembly accessions GCA_965278115.1 and GCA_965278145.1 are public. Earth BioGenome Project three-part metric: pass. Haplotype 1 is reported as 6.C.Q67, meeting the 6.C.Q40 reference standard. k-mer completeness: pass. The combined haplotype assemblies have 99.23% k-mer completeness, exceeding the Earth BioGenome Project target of 90%. BUSCO: pass. Haplotype 1, which is proposed as the reference assembly for this species, has 97.4% complete BUSCOs (92.4% single-copy and 5.1% duplicated). BUSCO software version: 5.7.1; lineage dataset: fabales_odb10. It meets the plant-specific target of greater than 90% complete BUSCOs. This decision uses values reported in the Genome Note and public accession records.

Reviewer Expertise:

NA

I confirm that I have read this submission and believe that I have an appropriate level of expertise to confirm that it is of an acceptable scientific standard.
